# The Use of Instrument-Assisted Soft-Tissue Mobilization for Manual Medicine: Aiding Hand Health in Clinical Practice

**DOI:** 10.7759/cureus.28623

**Published:** 2022-08-31

**Authors:** Luigi Pianese, Bruno Bordoni

**Affiliations:** 1 Physical Medicine and Rehabilitation, 3C+A Health and Rehabilitation, Rome, ITA; 2 Physical Medicine and Rehabilitation, Foundation Don Carlo Gnocchi, Milan, ITA

**Keywords:** musculoskeletal injury, thumb, pain, osteopathic, myofascial, fascia, work-related musculoskeletal disorders, iastm

## Abstract

Instrument-assisted soft-tissue mobilization (IASTM) represents a treatment strategy for soft tissue (skin) and musculoskeletal tissue (myofascia). There are different morphologies of these tools that are used by clinicians and manual therapists for the management of scars, fibrotic formations, muscle-joint pain, and movement limitations. The literature demonstrates the effectiveness of IASTMs in different clinical areas. However, the literature does not consider the use of these tools for the protection of the clinician’s hands. The main objective of this article is to draw attention to the fact that IASTM can protect clinicians from professional joint injuries of the hands and can likely become a preventive tool for the operator. Further research is needed to fully determine the positive adaptations in operators who use IASTMs compared to those who do not use them.

## Introduction and background

Injuries to the myofascia and skin in relation to competitive sports or recreational motor activities represent an important cause of hospitalization and care, with varying percentages depending on the anatomical area involved and the sport practiced. For example, hamstring injuries represent about 39% of all sports injuries, and groin injuries represent about 10-43% of the athletes involved in ice hockey [1,2]. One of the most common pathomechanisms of injuries is an eccentric contraction movement, which damages the contractile fibers and related connective tissues [3]. Another known cause is an overuse injury which can lead to partial or complete tissue injury, or create a chronic inflammatory environment, such as plantar fasciitis or pain that is not always clear to define clinically [4]. With the increase in soft-tissue injuries, the need to find curative strategies grows, which are not necessarily related to surgery. Among the non-surgical approaches, we can find manual medicine. To give some examples, manual therapy can be a valuable aid in osteoarthritis of the knee joint, as a recent review has shown. Osteopathy improves functional parameters in runners suffering from patellofemoral pain syndrome [5,6]. The chiropractic approach improves several musculoskeletal parameters in high-level athletes [7,8]. One of the tools of manual medicine used to implement soft-tissue function is instrument-assisted soft-tissue mobilization (IASTM) [9]. IASTM speeds up some parameters related to the healing processes of myofascial tissue and increases the musculoskeletal performance coefficients [9]. IASTM is manual instrumentation that derives from the therapeutic concepts introduced by James Cyriax. The digital cross-friction of the myofascia, which is a movement that allows the improvement of the blood supply [10]. Myofascial tissue comprises connective and contractile tissue [11]. The term IASTM appeared for the first time in an experimental search on PubMed in 1997 [12]. The study described the use of this instrument on an animal model, where a lesion on the Achilles tendon demonstrated a faster healing time. IASTM includes several hard material instruments (plastic or steel) with varied morphologies that the clinician can use manually depending on the depth of the applied pressure, or the clinical picture. These instruments are designed to exert longitudinal pressure along the path of the muscle and/or connective fibers [13]. The movement of the instrument, during the deformation of the soft tissues, generates vibrations; the latter allows the operator to discriminate a dysfunctional tissue compared to healthy tissue [14-16]. The pressures exerted by the instrument allow the operator to reach a greater tissue depth than using the hand alone [17]. The use of IASTM was positive in reducing the sensation of pain felt by the patient, in chronic (costochondritis) and acute (shoulder pain) musculoskeletal pathologies, with functional improvements [14,18,19]. In this article, we highlight the most important mechanisms related to the improvement of tissues after the application of the IASTM. The main objective of the review is to highlight the benefits of using these tools for protecting the joints of the operator’s/clinician’s hands, highlighting the most significant studies.

## Review

The evolution and physiological mechanism of IASTM

**Figure 1:**
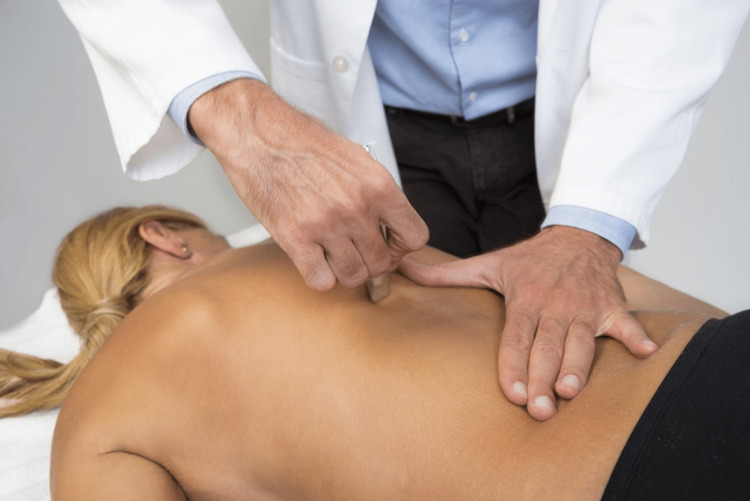
The use of an IASTM to soften some areas of the lower posterior thoracic area. IASTM: instrument-assisted soft-tissue mobilization Figure owned by Pianese Luigi.

**Figure 2:**
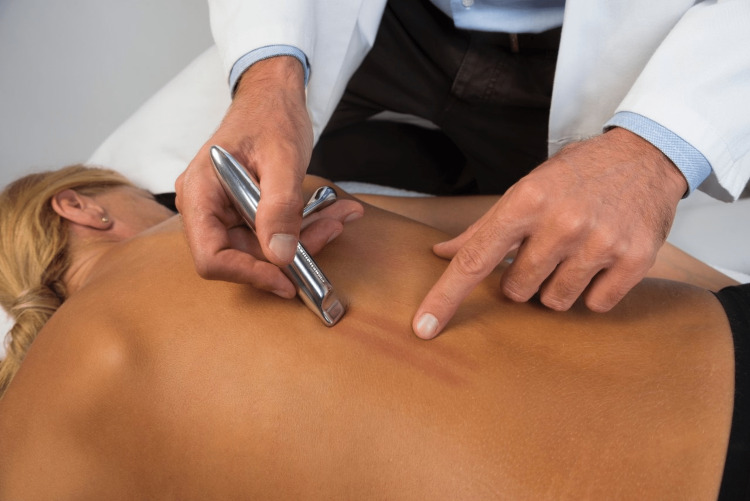
Skin hyperemia stimulated by the use of an IASTM. IASTM: instrument-assisted soft-tissue mobilization Figure owned by Pianese Luigi.

The need for instrumental application for therapeutic purposes dates back to Greek and Roman antiquity when small metal objects found wide application in therapeutic operations. Furthermore, such tools have also been mentioned by traditional Chinese therapists [4,20]. Instruments have often been used to scrape or squeeze the skin as a means of accelerating blood flow to facilitate the supply and delivery of oxygen and blood to the soft tissues [21]. The use of today’s IASTMs draws on the principle of using innovation and revisiting the Gua Sha of traditional Chinese medicine and the strigil used in ancient Greece for the mobilization of soft tissues. The IASTM tools are of various forms, and various techniques are associated with them, including fascial abrasion techniques, sound-assisted soft-tissue mobilization, and increased soft-tissue mobilization (Figures 1, 2) [9,14,22].

**Figure 3:**
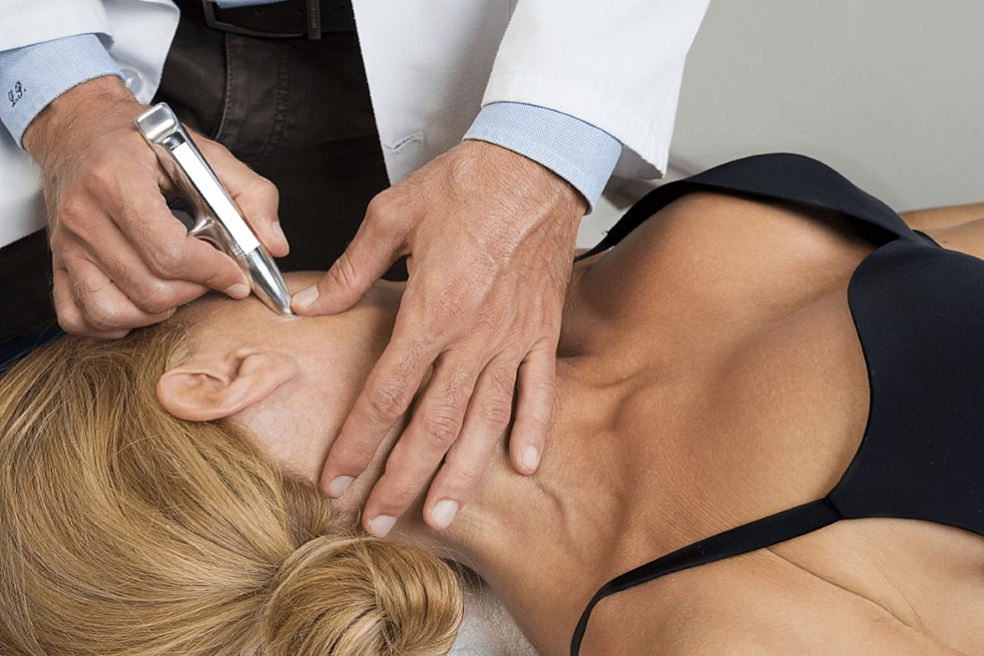
The delicate work of the clinician with an IASTM over the area of the masseter muscle. IASTM: instrument-assisted soft-tissue mobilization Figure owned by Pianese Luigi.

The names assigned to the various IASTMs depend on their shape and the various materials the instrument itself is made of, mainly stainless steel (as opposed to traditional skin stimulation tools which were made of wood or animal bones). Generally, IASTM is often applied as a simple and very practical technique by healthcare professionals [23]. When the clinic uses IASTM, the surface of the instrument that comes into contact with the affected area minimizes the force applied by the clinician and maximizes the amount of force exerted on the soft tissues. This practice facilitates the stimulation of deeper areas in delicate anatomical areas such as the face (Figure 3) [24,25].

A significantly high number of musculoskeletal soft-tissue injuries are the result of excessive tension and/or lack of adequate rest, especially among athletes and in sports [26,27]. A muscular environment in the presence of chronic inflammatory processes, for example, in the overtraining syndrome, can cause the formation of fibrosis [28]. The occurrence of such changes in the soft tissues causes a reduction in the elasticity of the tissue, resulting in adhesions that cause pain and exacerbate the deterioration of the functioning of the tissues [15,29]. The presence of scar tissue can lead to limited perfusion, depriving and limiting the supply of nutrients and oxygen [30]. Scar tissue interferes with collagen synthesis, inhibiting tissue regeneration [31]. Such a non-physiological context creates the tissue basis for a new possible re-injury [29]. The use of IASTMs facilitates and improves blood perfusion, promoting the reduction of symptoms of functional limitation related to the presence of scars and fibrosis [32]. The use of IASTM facilitates proper mobilization of fibroblastic cells and better deposit of collagen [12,14,21,25,33]. Numerous studies, both on animals and humans, have confirmed the results of this hypothesis. For example, a study involving rats treated using an IASTM targeting the treatment of enzyme-induced injury on the Achilles tendon showed a significant increase in the number of fibroblasts in tissue samples observed under a microscope [21]. The implication of these observations is that the treatment with IASTM stimulates the proliferation of fibroblasts, thus helping to accelerate the healing process of the tendons of the affected tissues; fibroblasts are directly linked to collagen synthesis [12,21]. Another study showed that early recovery of limb function in a rat model using IASTM was greatly facilitated by a possible mechanism attributable to angiogenesis, improving perfusion and vascularity in the area of injury [34]. The use of IASTM has made it possible to reduce the perception of pain in the inflamed myofascial tissue, improving motor function [35]. We do not know in detail what happens at the cellular level under the influence of the IASTMs, despite the benefits reported in the literature.

Pain and arthrosis of the thumb

**Figure 4:**
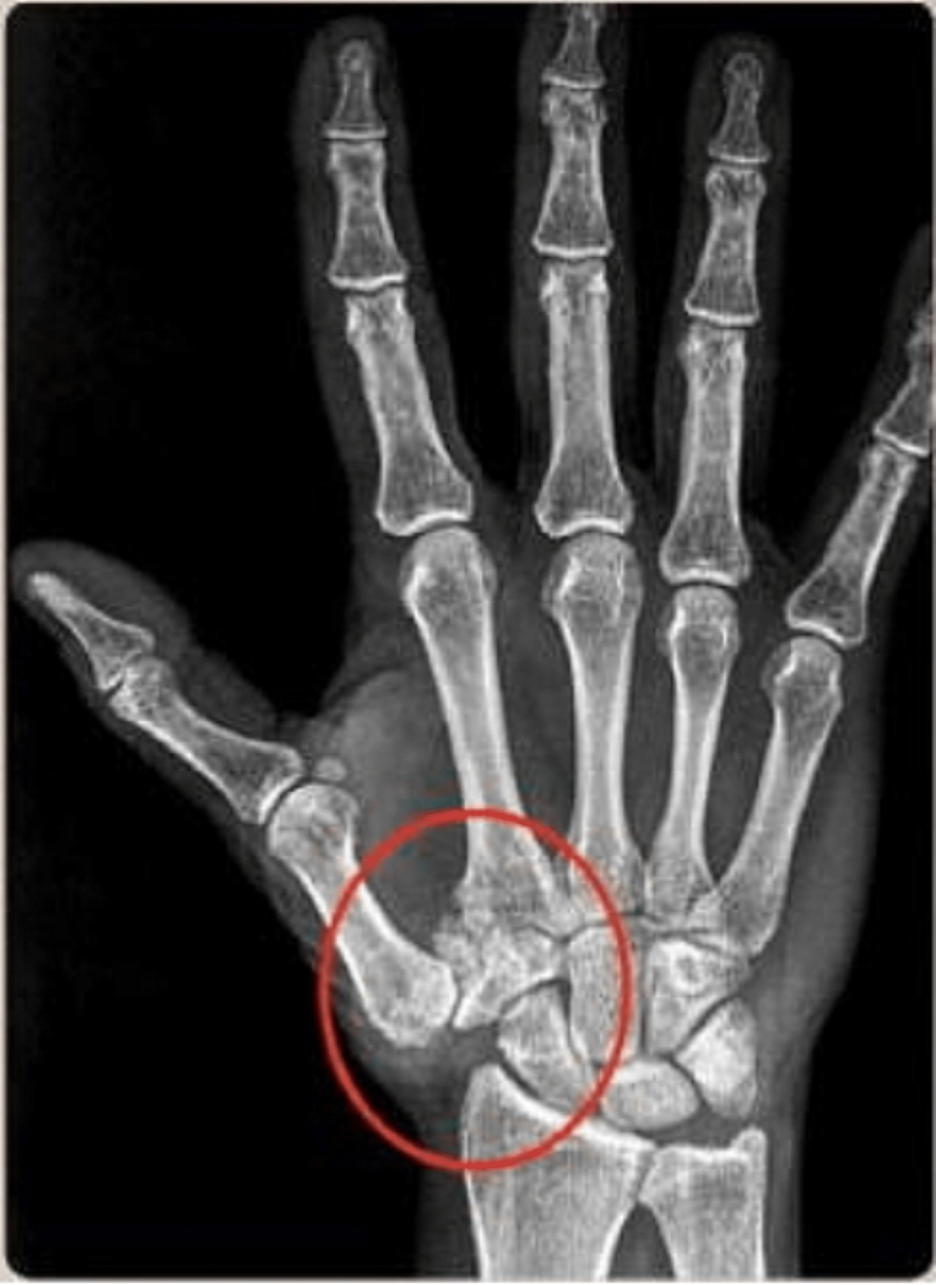
The plate of a hand highlighting the arthritic formation of the trapezium-metacarpal joint (circled area). Figure owned by Bordoni Bruno.

Manual therapists such as physiotherapists, physicians, chiropractors, and osteopaths are at increased risk and susceptibility to suffer the presence of musculoskeletal disorders [36]. In particular, the areas affected by overuse injuries are the trapezium-metacarpal joints and the joints of the hands in general (Figures 4) [37-40].

Manual injuries by operators can be harmful and disabling, with negative effects on the quality of life [41]. Manual techniques performed in a constant manner over the course of clinical activity can lead to pain in the hand and thumb. This event involves a negative change in the manual approach practiced and, at times, the clinician is unable to perform exhaustive work for the patient [40,42]. Based on the results of the survey through questionnaires administered to a cohort of 1,562 Australian physiotherapists, the prevalence of thumb problems was 65% (pain, hypomobility, hypermobility) [42]. Study participants cited a number of contributing factors to the onset of thumb problems. Among the common factors attributed, there is a direct relationship between manual work with patients with previous orthopedic surgery, different but constant manual techniques, massage, and manual therapy to reduce the symptoms of trigger points [42]. Most likely, a dysfunction of the hand could be the cause of further disorders of the upper limb (elbow and shoulder) [43]. The average thumb discomfort for manual work is more frequent between the ages of 50 and 70 [44].

The significant advantages of using an IASTM in manual therapy

**Figure 5:**
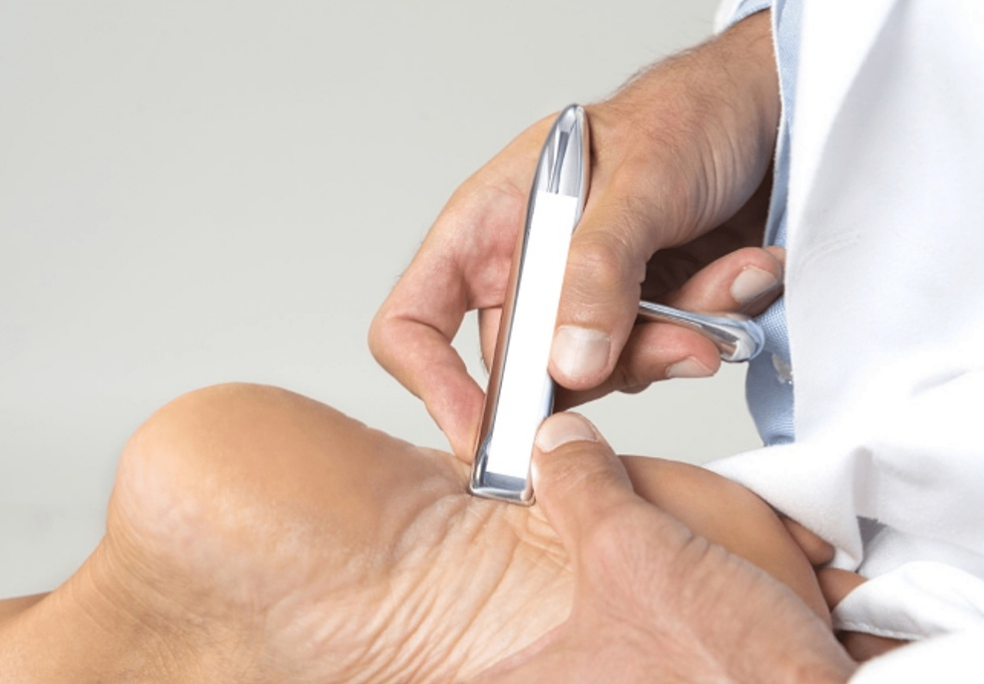
The use of an IASTM in a case of plantar fasciitis. IASTM: instrument-assisted soft-tissue mobilization Figure owned by Pianese Luigi.

The approach to improve the patient’s musculoskeletal dysfunctions, with the help of one or more IASTM, not only improves some functional parameters for the patient but can protect the joints of the operator’s/clinician’s hand. Recent studies have shown that clinicians who treat myofascial disorders can apply sufficient force to reach the desired tissue depth through the use of IASTM [45,46]. This allows the operator to save the use of the joints of the hand [47]. The use of the IASTM must be viewed from a multidisciplinary perspective, and its use can expand the possibilities for resolving the patient’s musculoskeletal dysfunction (Figure 5) [48,49].

**Figure 6:**
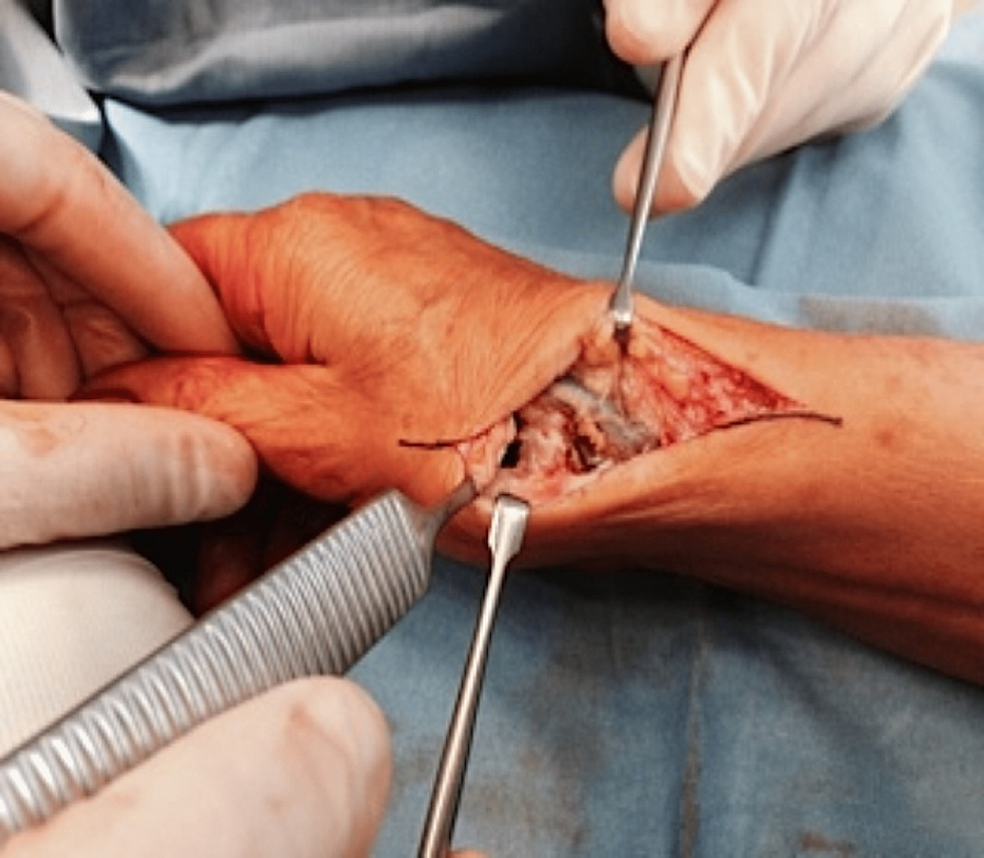
A phase of orthopedic surgery to eliminate peri-trapezius osteophytes, with exposure of the trapezius, along the incision centered on the trapezius-metacarpal joint. Figure owned by Bordoni Bruno.

IASTM offers a mechanical advantage to the clinician’s hand. By maintaining the same application force, the instrument can correctly follow the various morphologies of the patient’s anatomy [14]. This concept could result in less compression at the trapezius-metacarpal joint of the clinician. Sliding the IASTM over the patient’s skin or myofascial area (or a self-treatment) and perceiving different vibrations from the tissue allow the operator to identify denser and more painful areas, which are a sign of tissue suffering [15,16,24]. Applying less force when using the IASTMs means less neuromotor fatigue in the hand and fewer findings of injury to the clinician’s musculoskeletal system [24]. The operator could comfortably use the IASTM, despite the pain in the thumb, making the patient find the benefit more quickly [14,50,51]. If the clinician who uses manual therapy and who suffers from arthrosis of the trapezius-metacarpal joint can still work with the patient using IASTM, the time off work could be reduced (Figure 6) [40].

Further studies are needed to highlight all the benefits that the clinical operator who uses manual medicine can derive from the IASTM approach. Currently, the scientific literature highlights the symptomatological benefits following the use of IASTM for the patient; however, the literature is scarce regarding the positive adaptations deriving from the same instrument on the trapezium-metacarpal joint as well as on the joints of the hand for the clinician.

## Conclusions

This narrative review has highlighted some positive aspects of the symptomatological response of patients resulting from musculoskeletal injuries owing to the use of IASTM. These tools allow to speed up the recovery of myofascial and cutaneous tissues, stimulating some reparative and vascular processes that are not completely elucidated. The main objective of the article is to highlight the possible benefits for the clinician. The use of the IASTM allows the clinician not to overload their hand and thumb joints as the approach with manual medicine is one of the causes of injury that fall within occupational pathologies. Further research is awaited in this area to obtain more data on the musculoskeletal benefits that the operator can obtain from the use of IASTM.
